# Canine Mammary Cancer Stem Cells are Radio- and Chemo-Resistant and Exhibit an Epithelial-Mesenchymal Transition Phenotype

**DOI:** 10.3390/cancers3021744

**Published:** 2011-03-30

**Authors:** Lisa Y. Pang, Alejandro Cervantes-Arias, Rod W. Else, David J. Argyle

**Affiliations:** Royal (Dick) School of Veterinary Studies and Roslin Institute, The University of Edinburgh, Easter Bush, Midlothian, EH25 9RG, UK

**Keywords:** canine, breast cancer, cancer stem cell, drug resistance, TGFβ, EMT

## Abstract

Canine mammary carcinoma is the most common cancer among female dogs and is often fatal due to the development of distant metastases. In humans, solid tumors are made up of heterogeneous cell populations, which perform different roles in the tumor economy. A small subset of tumor cells can hold or acquire stem cell characteristics, enabling them to drive tumor growth, recurrence and metastasis. In veterinary medicine, the molecular drivers of canine mammary carcinoma are as yet undefined. Here we report that putative cancer stem cells (CSCs) can be isolated form a canine mammary carcinoma cell line, REM134. We show that these cells have an increased ability to form tumorspheres, a characteristic of stem cells, and that they express embryonic stem cell markers associated with pluripotency. Moreover, canine CSCs are relatively resistant to the cytotoxic effects of common chemotherapeutic drugs and ionizing radiation, indicating that failure of clinical therapy to eradicate canine mammary cancer may be due to the survival of CSCs. The epithelial to mesenchymal transition (EMT) has been associated with cancer invasion, metastasis, and the acquisition of stem cell characteristics. Our results show that canine CSCs predominantly express mesenchymal markers and are more invasive than parental cells, indicating that these cells have a mesenchymal phenotype. Furthermore, we show that canine mammary cancer cells can be induced to undergo EMT by TGF*β* and that these cells have an increased ability to form tumorspheres. Our findings indicate that EMT induction can enrich for cells with CSC properties, and provide further insight into canine CSC biology.

## 1. Introduction

Mammary tumors are the most common neoplasms that affect female dogs (*Canis familiaris*), constituting half of all tumors in female dogs and from these approximately half are considered malignant [1-3]. In both women and dogs, the incidence of mammary tumor development increases with age, rarely occurring before 25 and 5 years of age, respectively [4] and is hormone dependent [5]. Canine mammary carcinomas have epidemiologic, clinical, morphologic and prognostic features similar to those of human breast cancer and therefore represent a comparative model to understand the underlying molecular mechanisms of carcinogenesis in both species [4-6].

Recently, studies have identified subpopulations of cells within tumors that are responsible for tumor initiation, growth and metastasis; these cells have been termed cancer stem cells (CSC) [7,8]. CSCs have the capacity to self-renew, to initiate and maintain the tumor, and can produce heterogeneous lineages of cancer cells to compose the bulk of the tumor [9]. The cancer stem cell model therefore proposes that tumor development is akin to abnormal organogenesis [9]. In addition, CSCs are resistant to many current cancer treatments, including chemo- and radiation therapy [10-13]. Therefore conventional therapies, while killing the bulk of the tumor cells, ultimately fail because they do not eliminate the CSC population, which survives to regenerate the tumor. Further understanding the properties and mechanisms of CSCs is essential in the development of effective-anti-cancer therapies. In humans CSCs were first identified in acute myeloid leukemia [14], and more recently in melanomas [15,16], glioblastomas [17] and epithelial cancers [18-22]. In the canine model, we were the first to identify CSCs of a canine osteosarcoma cell line [23], and have subsequently isolated CSCs from a range of canine solid tumors including glioma, haemangiosarcoma and squamous cell carcinoma (data unpublished).

Recent evidence has suggested that tumor progression and metastasis is dependent upon aberrant activation of epithelial to mesenchymal transition (EMT) in cancer cells, resulting in the acquisition of invasive and metastatic properties [24]. Classically, EMT is an evolutionarily conserved developmental pathway involved in tissue morphogenesis, organ fibrosis and wound healing [25]. The hallmark of EMT is the loss of cell surface E-cadherin, which is associated with disassembly of adheren junctions, acquired motility and expression of mesenchymal markers including Vimentin and Fibronectin [26]. The EMT program is regulated by multiple transcription factors, including Twist, Snail and members of the ZFH family [27-29]. It is now known that EMT activation is also associated with the maintenance of stem cell properties, and *in vitro* it has been shown that emergence of CSCs occurs as a result of EMT [30-32].

In this study, we identified and characterized a subpopulation of putative CSCs from a canine mammary carcinoma cell line. Distinctive tumorsphere forming ability and expression of embryonic stem cell markers were identified in this subset and correlated with intrinsic resistance to DNA damaging drugs and ionizing radiation. This subset of putative CSCs was predominantly mesenchymal in terms of marker expression and invasive capacity. In addition we show, for the first time in canine cancer cells, TGF*β* induction of EMT and subsequent enrichment of cancer stem cells.

## 2. Material and Methods

### 2.1. Cell Culture and Tumorsphere Formation

Canine breast cancer derived REM134 cells (a kind gift from Prof. R.W. Else, The University of Edinburgh, UK) [33] were grown in Dulbecco's modified Eagle's medium (DMEM) (Invitrogen, Paisley, UK) supplemented with 10% fetal bovine serum and 100 μg/mL streptomycin (Invitrogen, Paisley, UK). For anchorage independent culture, REM134 cells were plated as single cells in ultralow attachment 6-well plates (Corning, CA, USA) at low cell density (1.5 × 10^4^ cells/mL). Cells were grown in serum-free conditional medium, which contained DMEM/F12 supplemented with progesterone (20 nM), putrescine (100 μM), sodium selenite (30 nM), transferring (25 μg/mL), insulin (20 μg/mL) (Sigma Biochemicals, Dorset, UK), human recombinant bFGF (10 ng/mL) and EGF (10 ng/mL) (Peprotech, NJ, USA). Additional growth factors (100 μg/mL) were added to the media every other day. All cell cultures were maintained at 37 °C in a humidified CO_2_ incubator.

### 2.2. Tumorsphere Forming Efficiency

The sphere forming ability of TGF*β* treated and untreated cells was determined by resuspending cells in serum-free conditional medium at a density of either 6000, 3000 or 1000 cells/well of 6-well low adherence plate (Corning, CA, USA). All experiments were conducted in triplicate. Plates were maintained at 37 °C in a humidified CO2 incubator and were maintained as before. After 7 days, the numbers of colonies were counted in 5 fields per well and representative views were photographed.

### 2.3. RNA Extraction and Reverse Transcription PCR Analysis

Total cellular RNA was extracted using RNeasy^®^ kit (Qiagen, CA, USA) and RNA quality was determined by A_260_ measurement. Semi-quantitative RT-PCR analysis of mRNA expression of stem cell specific genes including *Oct4*, and *Nanog* was performed using HotStar *Taq* polymerase (Qiagen, CA, USA) and the following specific primers:

*Oct4* sense 5′-CTCTGCAGCCAATCAACCACAA-3′
antisense 5′-GGAGAGGGGGATGAGAAGTACAAT-3′
*Nanog* sense 5′-CTATAGAGGAGAGCACAGTGAAG-3′
antisense 5′-GTTCGGATCTACTTTAGAGTGAGG-3′
*β-Actin* sense 5′-CATGTTTGAGACCTTCAACACCC-3′
antisense 5′-GCCATCTCTTGCTCGAAGTCCAG-3′

### 2.4. Irradiation and Drug Treatments of Cells

Cells were irradiated in culture media using a Faxitron^®^ cabinetX-ray system, 43855D (Faxitron X-ray Corporation, IL, USA), at a central dose of 2 Gy/min. Cells were irradiated at the stated doses. Cells were treated with Doxorubicin (Pfizer, Sandwich, UK) over the indicated range of concentrations. Cells were treated with 10 ng/mL Tgf*β* (Peprotech, NJ, USA) for the indicated times.

### 2.5. Protein Detection

Cells were lysed in urea lysis buffer (7 M urea, 0.1 M DTT, 0.05% Triton X-100, 25 mM NaCl, 20 mM Hepes pH 7.5). Equal amounts of protein were separated by SDS polyacrylamide gel electrophoresis (SDS PAGE), transferred to Hybond-C nitrocellulose membrane (Amersham Pharmacia Biotech, Buckinghamshire, UK) and hybridized to an appropriate primary antibody and HRP-conjugated secondary antibody for subsequent detection by ECL. Primary antibodies against β-actin and Vimentin were purchased from Abcam (Cambridge, UK). Antibodies against β-catenin, E-Cadherin and Fibronectin were purchased from BD Biosciences (Oxford, UK). Anti-Twist (L-21) was purchased from Santa Cruz Biotechnology (CA, USA). Secondary antibodies were HRP-conjugated rabbit anti-mouse IgG and swine anti-rabbit IgG, were obtained from DakoCytomation (Glostrup, Denmark).

### 2.6. Cell Viability Assay

REM134 cells were seeded in quadruplet in opaque 96-well plates (Corning, CA, USA) at 500 cells /well. A serial dilution of doxorubicin was added to the appropriate cells the following day. Alternatively, cells were treated with different doses of ionizing radiation. Dose-response curves were generated 72 hours after exposure. Cytotoxicity was measured using the CellTiter-Glo^®^ Luminescent Cell Viability Assay (Promega, Madison, USA), which quantifies the number of viable cells in culture based on quantification of ATP present. Data was averaged and normalized against the average signal of untreated/vehicle control treated samples.

### 2.7. Colony Formation Assay

Tumorspheres and corresponding adherent parental cells were trypsinised into single cells and seeded at 500 cells/10 cm plate. The cells were irradiated at 0 Gy, 1 Gy, 2.5 Gy and 5 Gy whilst in suspension. Plates were incubated at 37 °C in humidified CO_2_ incubator until colonies were visible. Growth media was changed once a week. The colonies were fixed by incubating with ice-cold methanol for 5 minutes at room temperature. Colonies were stained with Giemsa stain (Sigma-Aldrich, Dorset, UK) according to the manufacturer's instruction, and counted. Each experimental condition was assayed in quadruplicate.

### 2.8. Invasion Assay

The invasive ability of cells was determined using the QCM™ collagen-based cell invasion assay kit (Millipore, MA, USA) was used according to the manufacturer's instructions. Cells were seeded into the upper inserts at 1 × 10^5^ cells per insert in serum-free DMEM. Cells were incubated at 37 °C with 5% CO_2_ for 48 hours. None invading cells were removed. Cells that migrated through the gel insert to the lower surface were stained and quantified by colorimetric measurement at 560 nm.

### 2.9. Wound-induced Migration Assay

REM134 cells (1 × 10^6^) treated with either TGF*β* (10 ng/mL) or a vehicle control were seeded in 100 mm culture plates and cultured to at least 95% confluence. Monolayer cells were washed with media and then scrapped with a plastic 200 μl pipette tip. Cells were then incubated at 37 °C with 5% CO_2_. The “wounded” areas were photographed by phase contrast microscopy at 0, 4, 8, 24, 28, 32 and 48 hours after scraping. The relative migration distance was calculated by the following formula: Relative migration distance (%) = 100 (A-B)/A, where A is the width of the cell wound before incubation, and B is the width of the cell wound after incubation. Results are expressed as the mean ± standard deviation.

### 2.10. Statistical analysis

The results were presented as the mean ± SD. Data were analyzed using analysis of variance and a Student's t test. p values of <0.05 were considered significant.

## 3. Results

### 3.1. A Subpopulation of Canine Mammary Carcinoma Cells Have Tumorsphere-forming Capacity

**Figure 1.**
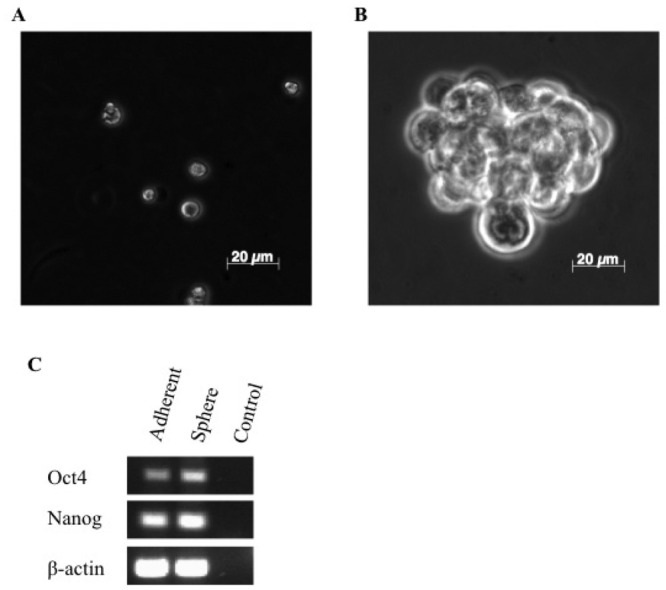
Isolation and characterization of putative cancer stem cells. Tumorsphere formation from the REM134 canine mammary carcinoma cell line. Single cells (A) and sphere (B). Non-quantitative RT-PCR analysis of mRNA expression of the embryonic stem cell markers *Oct4* and *Nanog* (C).

Previous studies have shown that cancer stem cells derived from a variety of human tumors tend to form spheroid colonies in defined serum free culture that favors the proliferation of undifferentiated cells [17,34,35]. Here, canine mammary carcinoma cells, REM134, were seeded as single cells at low-density into suspension cultures in serum-free growth factor supplemented media (Figure 1A). After 5–7 days tumorspheres were clearly visible (Figure 1B) and we estimated that approximately 1% of cells give rise to tumorspheres. To determine whether tumorspheres can be expanded *in vitro*, spheres were dissociated into single cell suspensions and passaged multiple times in long-term sphere forming assay. These cells repeatedly form tumorspheres for up to sixteen subsequent passages when plated under the stated culture conditions and in the absence of attachment.

To further characterize tumorspheres as a primitive sub-population of REM134 cells, we examined the expression of embryonic stem cell markers *Oct4* and *Nanog* Oct4 and Nanog are transcriptional determinants essential for self-renewal and maintenance of the undifferentiated state [36]. Here we show that *Oct4* and *Nanog* are expressed at a higher level in tumorspheres compared to parental adherent cells (Figure 1C). Thus, the canine mammary carcinoma cell line, REM134, contains a sub-population of cells that can survive in the absence of attachment, forms tumorspheres that can be expanded *in vitro*, and express embryonic stem cell makers which may be required for maintaining these cells in a primitive state.

### 3.2. Canine Mammary Carcinoma Stem Cells Exhibit Greater Resistance to Chemo- and Radiation Therapy

**Figure 2.**
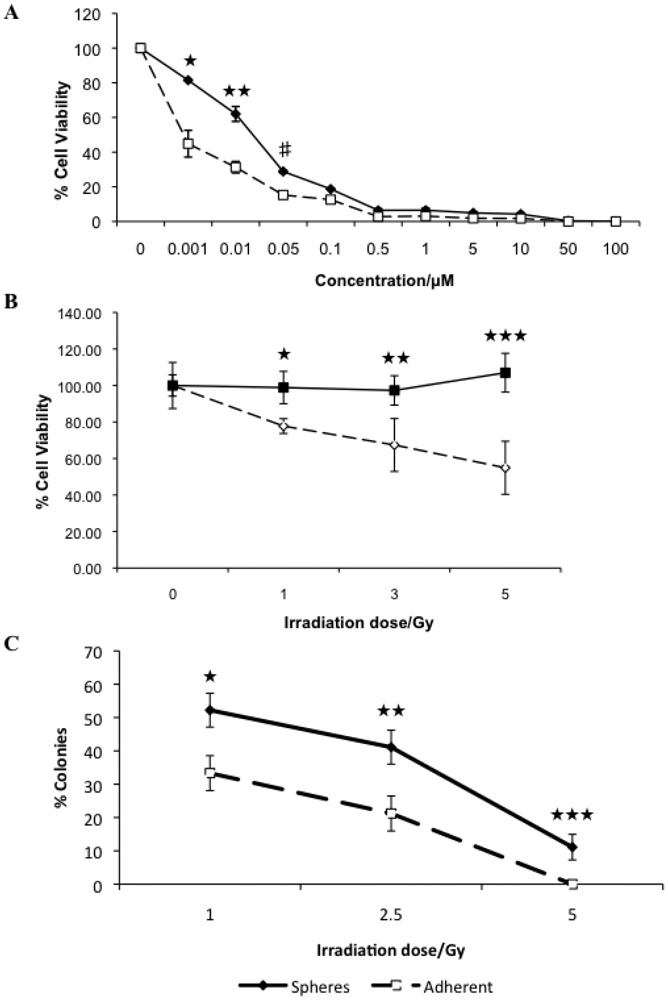
Tumorspheres exhibit increased resistance to conventional chemo- and radiation therapies. Adherent cells and tumorspheres were treated with increasing concentrations of doxorubicin and cell viability was assayed 72 hours post-treatment (★ *p* = 0.008; ★ ★ *p* = 0.038; #*p* < 0.001) (A). Radiation sensitivity was determined by assaying for cell viability 72 hours post-treatment (★ *p* = 0.003; ★ ★ *p* = 0.026; ★ ★ ★ *p* = 0.002) (B) and by determining colony forming ability (★ *p* = 0.01; ★ ★ *p* = 0.009; ★ ★ ★ *p* < 0.001) (C).

To determine whether tumorspheres cells preferentially survive after treatment with chemotherapeutic agents, tumorspheres were dissociated into single cells and treated with increasing concentrations of the cancer chemotherapeutic drug, doxorubicin. Doxorubicin is an anti-tumor antibiotic DNA damaging agent and is commonly used in veterinary and human cancer chemotherapy protocols. We used doses of Doxorubicin in cell culture experiments that correlate to concentrations that can be achieved *in vivo*. Cell viability was assayed 72 hours after treatment. Cells from tumorspheres demonstrated a significantly increased resistance to the cytotoxic effect of doxorubicin compared to parental adherent cells (Figure 2A).

Previously we have shown that tumorspheres derived from this cell line are more resistant to the anti-tumor effect of interferons- ω [37], consistent with increased resistance to doxorubicin. In addition, we compared the intrinsic radiosensitivity of cells dissociated from tumorspheres and parental adherent cells by measuring cell viability and clonogenic analysis. We used radiation doses based upon the therapeutic dose range. Adherent cells show a dose dependent decrease in cell viability, whereas tumorsphere viability is unaffected with increasing doses of ionizing radiation (Figure 2B). By colony formation assay we further confirmed that cells dissociated from tumorspheres are more resistant to ionizing radiation than corresponding adherent cells (Figure 2C). We have shown that REM134 cells with sphere-forming potential are more resistant to the therapeutic dose of DNA damaging agents and ionizing radiation *in vitro*, and therefore in a physiological setting may contribute to tumor repopulation.

### 3.3. Tumorspheres Display Mesenchymal Features and are More Invasive

**Figure 3.**
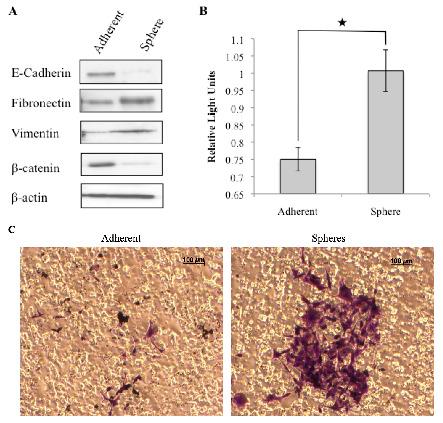
Putative cancer stem cells exhibit mesenchymal characteristics. Tumorspheres derived from the REM134 canine mammary carcinoma cell line express mesenchymal markers (A). Representative images of invading cells, stained purple, embedded within the membrane of a boyden chamber (C) and quantified by colorimetric measurement at 560 nm (★ *p* < 0.008) (B).

The metastatic process involves cell detachment from the extracellular matrix, migration from the tumor microenvironment and subsequent invasion and attachment at a secondary site within the body. Although the mechanisms underlying tumor cell invasion remain incompletely understood, EMT has been implicated by promoting loss of contact inhibition, increased cell motility and enhanced invasiveness [26,38]. Here we examined the expression of well-known epithelial markers (E-cadherin and β-catenin) and mesenchymal markers (Fibronectin and Vimentin) [39]. Tumorspheres were dissociated into single cells and compared to parental cells. Western blot analysis showed that the expression of E-cadherin and β-catenin was significantly decreased, whereas that of Fibronectin and Vimentin was significantly increased in tumorspheres compared to parental adherent cells (Figure 3A). Therefore tumorspheres that exhibit increased resistance to ionizing radiation and chemotherapy, have a mesenchymal phenotype.

As EMT is associated with increased invasiveness, the invasive capacity of cells dissociated from tumorspheres and matched adherent cells was evaluated using the Boyden chamber assay. Tumorspheres displayed a significantly greater invasive potential compared to adherent cells (Figure 3B and 3C), consistent with the hypothesis that cancer stem cells contribute to invasion and migration of the tumor.

### 3.4. TGFβ Treatment of REM134 Cells Induces an Epithelial to Mesenchymal Transition and Enhances Tumorsphere Forming Potential

**Figure 4.**
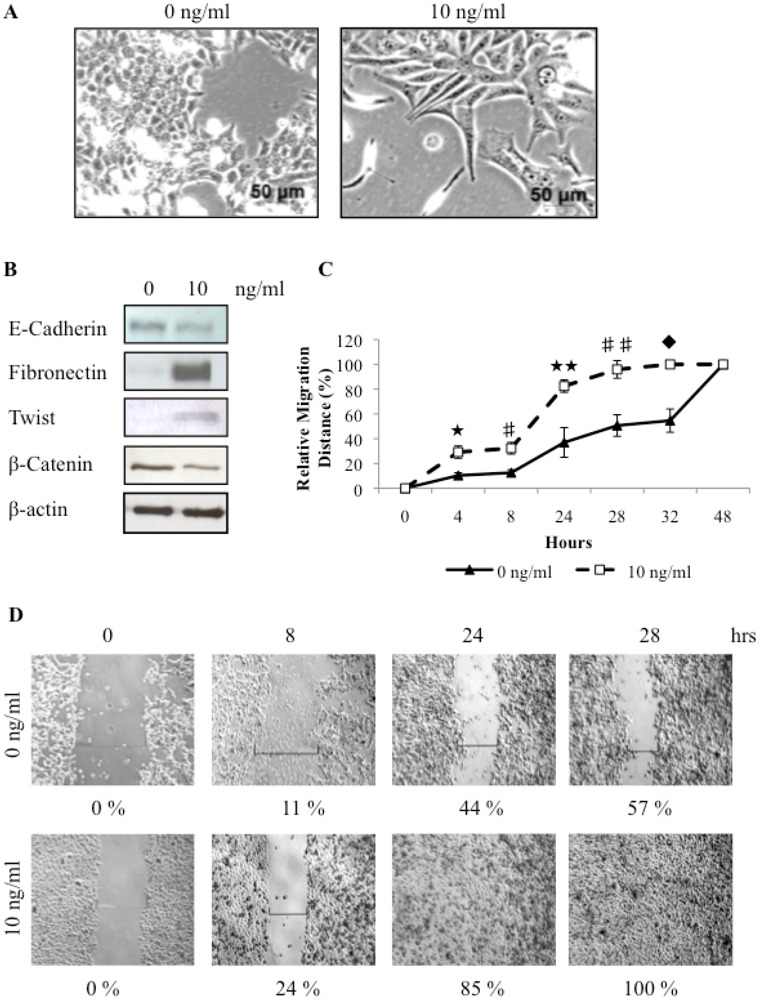
Treatment of canine mammary carcinoma cells with TGF*β* can induce an epithelial to mesenchymal transition, as indicated by changes in cell morphology (A), protein expression levels (B), and increased migration ability (★ *p* = 0.018; #*p* = 0.014; ★ ★ *p* = 0.004; ##*p* = 0.002; u*p* = 0.001) (C, D).

Previous studies have shown that EMT activation of human neoplastic mammary epithelial cells is associated with enrichment of cells with stem-like properties [31]. Here we have shown that canine tumorspheres have a mesenchymal phenotype and increased invasiveness, and may have undergone EMT. To investigate if an experimentally induced EMT in canine mammary carcinoma cells can also result in enrichment of putative cancer stem cells, we treated these cells with TGF*β*, a potent inducer of EMT. Within 72 hours of treatment with TGF*β*, the cells show a clearly manifested morphological change. The untreated cells are characterized by a cobblestone appearance whereas TGF*β* treated cells have an elongate fibroblastic phenotype indicative of mesenchymal cells (Figure 4A). The morphological change is associated with changes in protein expression of molecular markers of EMT. In the TGF*β* treated cells there is a decrease in the epithelial markers E-cadherin and β-catenin, and an up regulation of the mesenchymal markers Fibronectin and Twist (Figure 4B). This data supports the hypothesis that TGF*β* can activate EMT in canine cells.

To investigate the effect of TGF*β* on cellular migration, monolayer wound-induced migration assays were performed. A wound was made in a sub-confluent cell monolayer and cells were allowed to migrate into the cell-free area. The distance moved by cells in the untreated and TGF*β* treated cells was compared. TGF*β* treatment significantly enhanced the migration and wound healing capacity of REM134 cells (100% closure of the wound in 28 hours) as compared to untreated cells (57% closure in 28 hours) (Figure 4C and D). Thus, the migratory potential of REM134 cells is enhanced by TGF*β* treatment.

**Figure 5.**
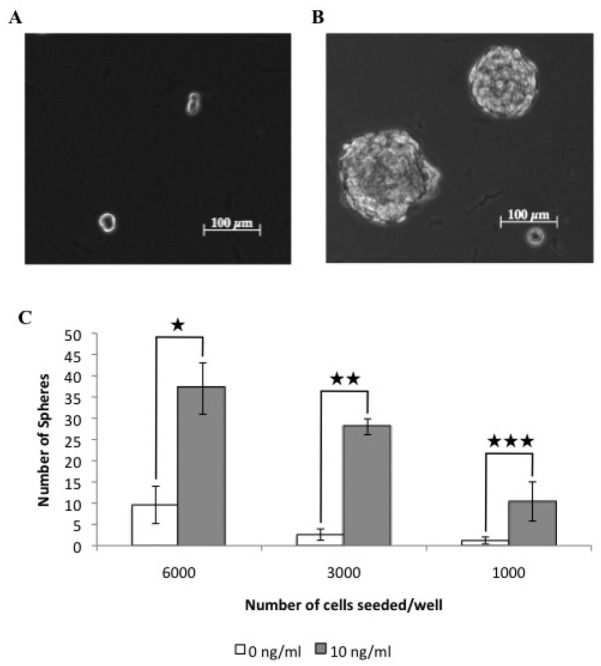
TGF*β* treated cells show an increased tumorsphere forming ability compared to untreated cells. Tumorspheres resulted less frequently from untreated cells (A), compared to cells treated with 10 ng/ml TGF*β* (B). The resultant number of spherical colonies were counted (★ *p* < 0.001; ★ ★ *p* < 0.001; ★ ★ ★ *p* < 0.01) (C).

EMT activation is proposed to enrich the proportion of cancer stem cells with a given cell population. Therefore we tested the ability of TGF*β* treated cells to form tumorspheres when grown in suspension cultures, as an *in vitro* measure of cancer stem cell activity. TGF*β* treated cells formed large clearly identifiable tumorspheres after 7 days in culture (Figure 5A) and showed an ∼8-fold increase in tumorsphere forming ability relative to untreated cells (Figure 5B). This data indicates that canine mammary carcinoma cells induced to undergo an EMT by TGF*β* contained a significantly greater proportion of cells with a CSC-like phenotype compared to control cells.

## 4. Discussion

Breast cancer is a major cause of morbidity and mortality in women. However, the majority of cancer related therapeutic studies rely upon rodent models of human cancer that rarely translate into clinical success in human patients [9,40]. Recent advances in veterinary medicine, notably vaccination regimes for once fatal infectious diseases, has led to an increase in age-related diseases of our pet dogs and cats which mirrors the pattern of human public health. In the UK, approximately 1 in 3 dogs will develop cancer, and with approximately 7 million dogs resident in the UK, this provides an exciting opportunity to exploit these cancers in terms of identifying cancer-associated genes, identifying environmental risk factors and understanding tumor progression [9]. In contrast to rodent models of human cancer, cancers that arise in dogs develop naturally and in the context of an intact immune system where tumor, microenvironment and host are syngeneic. Histologically, the majority of human cancers are well represented in the canine population, including breast cancer, melanoma, head and neck squamous cell carcinoma and osteosarcoma, and follow a similar clinical course [41]. Importantly, these observed similarities can be supported with genetic evidence, with the publication of the canine genome and the increased portfolio of molecular tools available for this species [42]. For example, a recently constructed syntenic karyotype map between humans and dogs demonstrated strong similarities in cytogenetic abnormalities in Non-Hodgkin lymphoma occurring in both these species [43].

With regards to breast cancer, the gene expression profile of metastatic canine mammary carcinomas has been determined by utilizing the canine specific affymetrix array. Importantly, this expression profile significantly overlaps with expression profiles of metastatic human breast cancer. In the subset of overlapping genes there is enrichment of genes associated with cell cycle regulation, protein kinases, DNA integrity checkpoint and protein metabolism [44]. In humans, several genes predisposing to breast cancer have been identified, but the majority of risk factors remain unknown. Even less is known about the inherited risk factors underlying canine mammary tumors. Germ line mutations in BRCA1 and BRCA2 account for 5% to 10% of all breast cancer in women [45], and correspondingly in dogs, germ line mutations in the same genes, as determined by candidate gene association, have also been shown to predispose to canine mammary carcinoma [46]. This recent evidence indicates that the molecular drivers and mechanisms of canine and human carcinogenesis are analogous. Here we further contribute to the evidence that canine mammary carcinoma is a model system that can be used alongside traditional rodent models to study the equivalent human disease. In the present study we utilized the REM134 cell line, and report that a subpopulation of canine mammary carcinoma cells may be representative of canine cancer stem cells. Putative CSCs were characterized by their tumorsphere forming capacity, expression of embryonic stem cell markers and resistance to chemo- and radiation therapy. These results are comparable to the human model of mammary carcinoma. In future studies we intend to evaluate the *in vivo* tumorigenic potential of isolated canine CSCs, and to investigate if CSCs can be isolated from primary canine breast cancer tissue.

Breast cancer was the first solid tumor from which CSCs were isolated, and this seminal paper provided compelling evidence for the presence of functional heterogeneity within the tumor population [18]. CSCs exhibit an ability to undergo self-renewal while sustaining a multipotent differentiation capacity to maintain tumor development indefinitely [7,47]. Previous studies have also shown that CSCs can be isolated from established cell lines, indicating that they are also maintained in a cellular hierarchy [48,49]. Human breast cancer stem cells were initially isolated by high expression of CD44 and low expression of CD24[18]. We have characterized a diverse range of established canine cancer cell lines, including REM134 mammary carcinoma, D17 osteosarcoma, J3T glioma, SB haemangiosarcoma, 3132 B-cell Non-Hodgkins lymphoma, and have determined that expression of CD44 in canine cancer cells is cell cycle dependent (data unpublished) and is therefore not representative of a CSC marker in this species. However, sphere formation is also an established technique used to enrich stem cells [17], and here we show that a subpopulation of canine mammary carcinoma cells can promote clonogenic tumorsphere formation and be serially passaged for multiple generations. We further characterized tumorspheres using the embryonic stem cell markers, Nanog and Oct4. Constitutive expression of the transcription factor Nanog maintains the stem cell phenotype, allowing for self-renewal and propagation [50]. Similarly, Oct4 is a POU family transcription factor, which is initially expressed in the inner cell mass of the embryo and is essential for the maintenance of pluripotency [51]. We found that canine tumorspheres express higher levels of the *Oct4* and *Nanog* compared to parental cells. This data supports that tumorspheres have a primitive phenotype and are representative of a CSC population.

After surgery, ionizing radiation and chemotherapy are the most effective therapy for treating both human [52] and canine mammary carcinomas, however their effectiveness remains only palliative because of the ultimate development of treatment resistance [53,54]. The CSC theory proposes that CSCs are inherently resistant to conventional therapies and have the ability to repopulate the tumors after treatment [11]. Our results confirmed that tumorspheres derived from a canine mammary carcinoma cell line are more resistant to the chemotherapeutic drug doxorubicin and ionizing radiation, compared to parental cells. The molecular mechanisms underlying the intrinsic resistance of CSCs to conventional therapies remain elusive. However, previous studies have shown that CSCs express high levels of drug transporters [55,56]; anti-apoptotic proteins [57,58]; and preferentially activate DNA damage pathways [10]; Wnt/β-catenin pathways [59] and Akt/PKB survival pathways [60]. Interestingly, EMT of tumor cells has also been shown to contribute to drug resistance [25,30,61,62], although this remains mechanistically undefined.

Compelling evidence exists relating EMT to tumor metastasis and more recently to the emergence of CSCs. EMT is classically associated with the process of tissue morphogenesis during embryonic development, and is characterized by complex changes in gene expression [24]. Thus, epithelial and mesenchymal cells can be clearly distinguished by the expression of a number of markers [24]. EMT during embryonic development involves the loss of polarity and gain of motile characteristics of mesenchymal cells, which has prompted comparisons with metastatic cancer cells during malignant progression [63]. Subsequent studies have shown that EMT is a key step towards cancer metastasis, and that induction of EMT enhances cancer metastasis through enhanced invasion [64]. Here, we characterised tumorspheres and parental adherent cells in the context of EMT markers. We clearly show that tumorsphere cells have a mesenchymal phenotype, including down regulation of epithelial markers E-cadherin and β-catenin, and up regulation of mesenchymal markers Fibronectin and Vimentin, compared to parental adherent cells. Significantly, we also show that tumorspheres are more invasive than parental adherent cells, which is also indicative of a mesenchymal phenotype. Our findings are coherent with human breast cancers and breast cancer cell lines which display features of EMT [65,66], and with recent studies which have reported that loss of E-cadherin expression is positively correlated with advanced histological grade, metastasis and decreased survival [67,68].

Induction of EMT in normal or neoplastic mammary epithelial cells, by TGF*β* treatment or siRNA-mediated inhibition of the human *CDH1* gene that encodes E-cadherin, has been shown to result in the enrichment of cells with stem-like properties [31,32]. This observation has enabled, for the first time, high-throughput screening to identify compounds with specific activity against CSCs [30]. To our knowledge, we are the first to show that TGF*β* treatment of canine mammary carcinoma cells can induce a change in cell morphology, expression of EMT markers and increased invasiveness consistent with induction of an EMT. Significantly, we also show that TGF*β* treated canine mammary carcinoma cells have enhanced sphere-forming ability, indicating that EMT induction can enrich for canine cancer stem cells. CSC populations typically comprise only small minorities of cancer cell populations, typically less than 1% [69,70]. Our data confirms that canine CSC populations can be enriched *in vitro* by induction of EMT. This may enable us to further elucidate the mechanisms of CSC biology in the context of epigenetic regulation, drug and radiation resistance, and metastasis.

## 5. Conclusions

In summary, our data demonstrates that a putative cancer stem cell population can be isolated from a canine mammary carcinoma cell line. We show that these cells express embryonic stem cell markers, are invasive and are inherently resistant to radiation and chemotherapy. Significantly, we show that EMT induction can enrich the cancer stem cell population, and may be exploited to evaluate novel pathways to be targeted to increase therapeutic response in a clinical setting. Our results are consistent with human breast cancer models, and ultimately support the use of companion animals as a pre-clinical model system.
