# Correction: Pang et al. Canine Mammary Cancer Stem Cells Are Radio- and Chemo- Resistant and Exhibit an Epithelial-Mesenchymal Transition Phenotype. *Cancers* 2011, *3*, 1744–1762

**DOI:** 10.3390/cancers14174242

**Published:** 2022-08-31

**Authors:** Lisa Y. Pang, Alejandro Cervantes-Arias, Rod W. Else, David J. Argyle

**Affiliations:** Royal (Dick) School of Veterinary Studies and Roslin Institute, The University of Edinburgh, Easter Bush, Midlothian EH25 9RG, UK

## Error in Figure

In the original article [1], there was a mistake in Figure 3C as published. There was an image duplication. The corrected Figure 3C appears below. 

**Figure 3:**
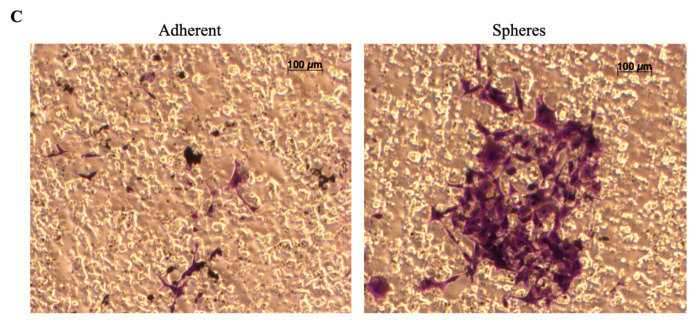
(**C**). Representative images of invading cells, stained purple, embedded within the membrane of a boyden chamber.

The authors apologize for any inconvenience caused and state that the scientific conclusions are unaffected. 
